# Application of photo-crosslinkable gelatin methacryloyl in wound healing

**DOI:** 10.3389/fbioe.2023.1303709

**Published:** 2023-11-23

**Authors:** Jinli Zhang, Changling Liu, Xiaojian Li, Zhihe Liu, Zhi Zhang

**Affiliations:** ^1^ Guangzhou Institute of Traumatic Surgery, Guangzhou Red Cross Hospital (Guangzhou Red Cross Hospital of Jinan University), Guangzhou, China; ^2^ Department of Burns and Plastic Surgery, Guangzhou Red Cross Hospital (Guangzhou Red Cross Hospital of Jinan University), Guangzhou, China

**Keywords:** gelatine methacryloyl, wound healing, skin tissue engineering, hydrogels, wound dressing

## Abstract

Wound healing is a complex and coordinated biological process easily influenced by various internal and external factors. Hydrogels have immense practical importance in wound nursing because of their environmental moisturising, pain-relieving, and cooling effects. As photo-crosslinkable biomaterials, gelatine methacryloyl (GelMA) hydrogels exhibit substantial potential for tissue repair and reconstruction because of their tunable and beneficial properties. GelMA hydrogels have been extensively investigated as scaffolds for cell growth and drug release in various biomedical applications. They also hold great significance in wound healing because of their similarity to the components of the extracellular matrix of the skin and their favourable physicochemical properties. These hydrogels can promote wound healing and tissue remodelling by reducing inflammation, facilitating vascularisation, and supporting cell growth. In this study, we reviewed the applications of GelMA hydrogels in wound healing, including skin tissue engineering, wound dressing, and transdermal drug delivery. We aim to inspire further exploration of their potential for wound healing.

## 1 Introduction

The skin is a multifunctional barrier organ that protects internal organs from potential environmental hazards (Lee et al., 2006). The protective function of the skin’s barrier can be damaged by conditions such as burns, trauma, diabetes, and local pressure effects. Skin wound healing is an ordered and complex biological process that primarily includes haemostasis, inflammation, proliferation, and remodelling (Rahimnejad et al., 2017). However, this wound healing process may be interrupted and altered because of conditions such as diabetes, renal disease, lower immunity, and advanced age. These factors generally lead to delayed wound healing because of insufficient blood supply and wound infection (Eming et al., 2014). Furthermore, it is almost impossible for the skin to heal properly when skin defects are too large (Fu et al., 2023). Several therapies have been developed for addressing delayed wound healing, including vacuum-assisted closure, stem cell therapy, and biological dressings. Hydrogel dressings are widely used in wound nursing because of their good biocompatibility, moisture retention, and drug delivery performance (Qiu and Park, 2001; Khademhosseini and Langer, 2007; Banerjee et al., 2018). In contrast to traditional dressings, hydrogel dressings can not only absorb wound exudates and maintain the moist environment of the wound but can also accelerate wound healing upon loading with various biological components and cytokines. In recent years, GelMA hydrogels have attracted increasing attention as artificial extracellular matrix (ECM) materials for wound healing. GelMA hydrogels have broad prospects for skin wound healing owing to their excellent biocompatibility and tunable mechanical properties (Xu et al., 2019; Leu Alexa et al., 2021; Kurian et al., 2022).

**FIGURE 1:**
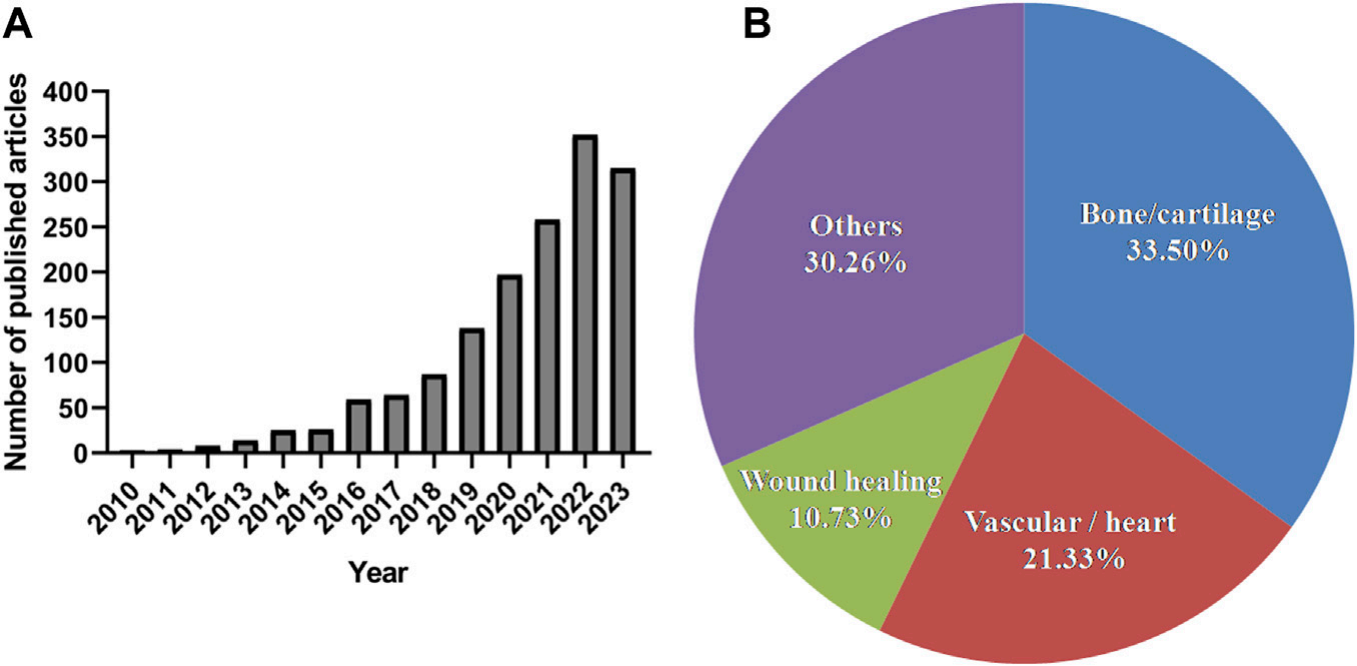
The number of published articles related to GelMA and their research fields during the last 10 years according to the PubMed (report acquired on 14 August 2023 using advanced keyword search; keywords: GelMA; “bone or cartilage”; vascular; “wound healing”; cardiac).

GelMA is a synthesised biomacromolecule with excellent biocompatibility and formability; it was first reported to be synthesised by Bulcke et al., in 2000 (Van Den Bulcke et al., 2000). Since then, an increasing number of studies have focused on this photocurable multifunctional biomaterial (Figure 1A). We performed a simple data analysis of these published research articles and found that they were primarily concentrated in the fields of bone and cartilage (33.5%), vasculature and heart (21.33%), and wound healing (10.73%) (Figure 1B). GelMA can serve as a versatile matrix for bone and cartilage tissue engineering scaffolds. It does so by introducing inorganic composites, growth factors, and even cells to mimic the structural, mechanical, and biological properties of natural bone and cartilage (Goto et al., 2021; Dong et al., 2019). Many studies have proven that vascular network structures can be formed by embedding human vascular endothelial and mesenchymal cells into GelMA hydrogels (Chen et al., 2012; Nikkhah et al., 2012). GelMA hydrogels loaded with vascular cells can be microfabricated using different methods and used for disease modelling or drug screening via integration with microfluidic devices (Liu et al., 2020; Kinstlinger and Calderon, 2021). In addition to their use as tissue engineering scaffolds, their attractive properties are widely employed in the manufacturing of adhesives, wound dressings, and drug delivery carriers (Kulkarni et al., 2022; Kurian et al., 2022; Li et al., 2023a).

GelMA has significant advantages in skin repair and regeneration. As a gelatine derivative, GelMA is an ideal biomaterial candidate for engineering skin tissues because of its similarity to ECM and its tunable physical and chemical properties (Anand et al., 2022). With the development of 3D printing technology, GelMA is generally used as an excellent bioprinting ink for fabricating tissue-engineered skin (Leu Alexa et al., 2021). GelMA hydrogels can be used to simulate native tissues by controlling and designing various microstructures, which provides an ideal platform for tissue engineering (Zhang et al., 2022). Furthermore, GelMA hydrogels are suitable dressings for skin wound healing. In addition to maintaining a moist and clean environment for wounds, the *in situ* photo-crosslinking properties make GelMA hydrogels particularly suitable for application in irregular wounds (Wang et al., 2023). Importantly, GelMA hydrogels can promote wound healing by controlling bleeding, reducing inflammation, facilitating vascularisation, and accelerating collagen deposition via the encapsulation and sustained release of drugs (Im and Lin, 2022; Zhu et al., 2022; Xiong et al., 2023). Because of their unique ability to fill and adapt to irregular wounds, they are also used as haemostatic materials. In particular, the injectability and porous structure of GelMA hydrogels make them attractive for use in haemostasis (Chang et al., 2021; Wang et al., 2021). Therefore, GelMA is a promising biomaterial for skin tissue engineering and wound dressing. Here, we summarise the role of GelMA in skin tissue engineering and wound dressing and hope to provide valuable inspiration for the practical application of GelMA in wound healing.

## 2 Synthesis and biological properties of GelMA

**FIGURE 2:**
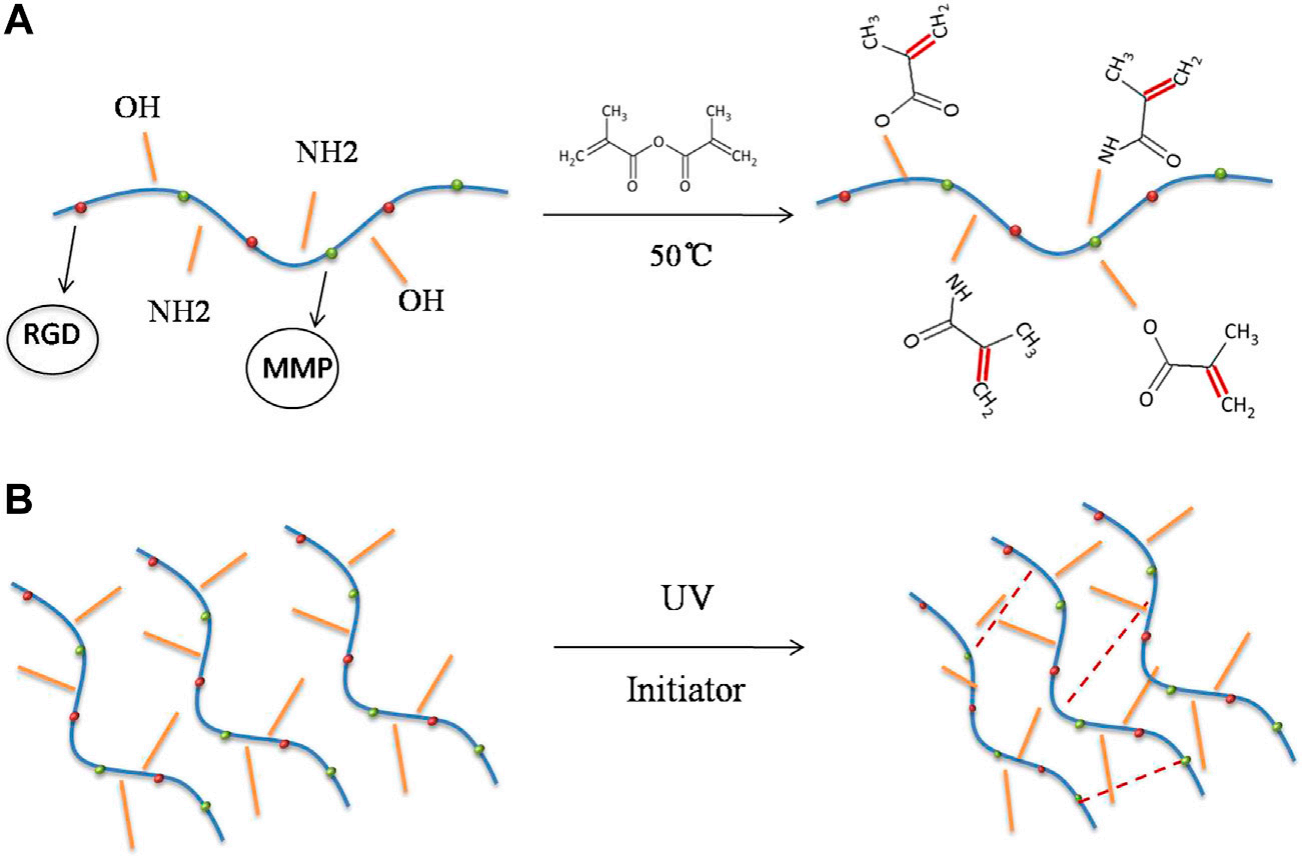
Synthesis of gelatine methacrylate (GelMA) hydrogel. **(A)** The amine (–NH_2_) and hydroxyl (–OH) groups on the side chains of gelatine are substituted by the methylacryloyl group at 50°C. **(B)** Crosslinking reaction of GelMA hydrogel initiated by UV radiation.

GelMA is produced via the reaction of gelatine and methacrylic anhydride, in which the amine (–NH_2_) and hydroxyl (–OH) groups on the side chains of gelatine are substituted by the methylacryloyl group (Figure 2A) (Yue et al., 2015). In the presence of a photoinitiator, the aqueous GelMA prepolymer solution was cross-linked to form hydrogels under ultraviolet (UV)-visible light irradiation (Figure 2B) (Yue et al., 2015). Gelatine is a hydrolysis product of collagen and has a wide range of biomedical applications because of its good biocompatibility and biodegradability (Sung et al., 1999; Liu et al., 2015). It is also widely used as an adhesive, thickener, emulsifier, and stabiliser in the food industry (Djagny et al., 2001; Ali et al., 2018). Gelatine has also been utilised in tissue engineering, cell encapsulation, and drug delivery (Olsen et al., 2003; Klotz et al., 2016). Unmodified gelatine forms physical cross-links only at a specific concentration and temperature, resulting in poor mechanical properties of the gelatine (hydrogel). However, the mechanical properties of GelMA hydrogels can be precisely adjusted based on the degree of methacrylate substitution and the time and intensity of light exposure (Yue et al., 2015). At the same time, the functional amino acid motifs of gelatine are not significantly influenced, because less than 5% of the amino acid residues in gelatine are generally modified (Van Den Bulcke et al., 2000). GelMA contains many arginine-glycine-aspartic acid and matrix metalloproteinase sequences such as those of gelatine and is suitable for cell attachment and remodelling (Benton et al., 2009; Nichol et al., 2010). Upon methacryloyl modification, GelMA not only exhibits additional photo-crosslinking and tunable properties but also maintains the original excellent biocompatibility of gelatine.

## 3 Application of GelMA in wound healing

**FIGURE 3:**
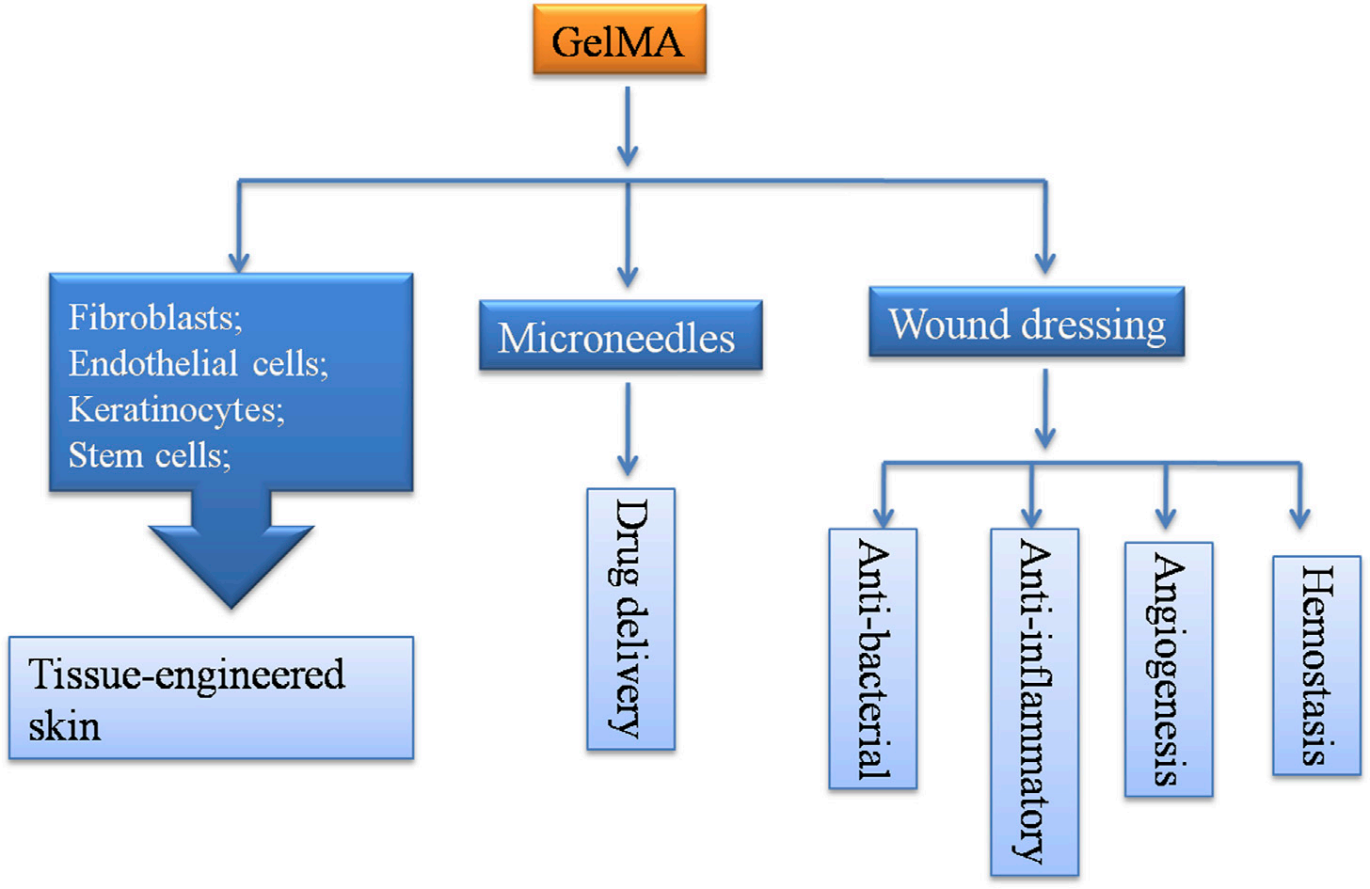
Potential applications of GelMA in wound healing.

Poor wound healing is generally associated with poorly regulated aspects of the normal tissue repair responses such as inflammation, angiogenesis, and cell recruitment (Gantwerker and Hom, 2011; Hom and Davis, 2023). GelMA is a promising material for wound treatment because of its structural similarities to the ECM and its multifunctional characteristics. GelMA serves as a versatile material for constructing tissue-engineered skin when combined with other natural ECMs, synthetic materials, or seed cells (Xiao et al., 2019; Kurian et al., 2022). The cell-interactive properties of GelMA hydrogels can stimulate the granulation tissue formation (Jahan et al., 2019; Do Nascimento et al., 2023). Furthermore, GelMA hydrogels provide a suitable environment for vascular morphogenesis, which is beneficial for wound vascular network formation (Im and Lin, 2022). Additionally, GelMA hydrogels can ideally absorb wound exudates and blood because of their strong water-uptake capacity (Baghdasarian et al., 2022; Guo et al., 2022). In this study, we propose several potential applications of GelMA in wound healing, as shown in Figure 3.

### 3.1 The applications of GelMA in fabricating tissue-engineered skin

Scaffold materials in skin tissue engineering can serve dual purposes: providing a scaffold to promote and guide cell proliferation and differentiation and creating an environment containing signalling substances and nutrients to achieve the desired biological characteristics (Khademhosseini et al., 2006; Burdick and Vunjak-Novakovic, 2009). GelMA, a derivative of gelatine, is a potentially attractive material for tissue engineering applications because it is an inexpensive and abundantly denatured collagen. GelMA has been implicated in regulating the growth of various cells, including fibroblasts, vascular endothelial cells, and keratinocytes and is widely used as a scaffold from simple cell culture to 3D culture (Table 1). As a cell-responsive hydrogel platform, cells either seeded on a micro-patterned GelMA hydrogel matrix or encapsulated in micro-manufactured GelMA hydrogels exhibited a tendency to adhere, proliferate, elongate, and migrate (Benton et al., 2009; Nichol et al., 2010). Additionally, the versatility of GelMA hydrogels is a significant advantage. Supplementing GelMA hydrogels with other scaffolds improved the properties of the scaffold materials and cell spreading. GelMA can be mixed with other methacrylated materials to form photo-crosslinkable composite hydrogels. For example, to improve the mechanical properties of a decellularised human amniotic membrane (dHAM), Zhang et al. fabricated a photo-crosslinkable composite hydrogel by grafting HAM with methacrylic anhydride and blending it with GelMA. According to the results of the tensile test, the maximum load value of GelMA-dHAMMA hydrogels was 2,430 ± 91 Pa, which was significantly higher than that of GelMA, dHAM, and dHAMMA (*p* < 0.05). GelMA-dHAMMA hydrogels were found to promote fibroblast proliferation and α-smooth muscle actin expression. GelMA-dHAMMA has also been shown to promote wound collagen deposition and angiogenesis and accelerate tissue healing in rabbit full-thickness skin tissue defects (Zhang et al., 2021). To simulate the structure of the skin tissue, Shen et al. constructed a tissue-engineered skin model with a rete ridge (RR) microstructure using 10% GelMA and 2% poly (ethylene glycol) diacrylate (PEGDA) (Shen et al., 2021). PEGDA (2%) was added to the GelMA prepolymer solution to slow the biodegradation rate and improve mechanical stability. First, they designed and fabricated a polydimethylsiloxane (PDMS) mould with a three-dimensional RR microstructure, into which the GelMA-PEGDA prepolymer solution suspended with HSFs was poured; it was then cross-linked by near-UV blue light to form GelMA-PEGDA hydrogel. Subsequently, HaCaTs were seeded on the GelMA-PEGDA hydrogel and cultured at the air-liquid interface to mimic the native skin tissue structure. Digital microscopy image analysis showed that the micropatterns were well-transferred to the scaffold’s surface. The fluorescence micrograph of the skin model section showed that HSFs and HaCaTs grew in their respective spaces and showed an undulating growth state. Additionally, epidermal cells and fibroblasts on the hydrogel scaffolds maintained proliferation and differentiation and promoted wound healing *in vivo*.

**TABLE 1:** Applications of GelMA in tissue-engineered skin and regeneration.

Composite components	Seed cells	Property
dHAM	Fibroblasts	➢ Promote large-area or full-thickness skin defect healing Zhang et al. (2021)
—	ESCs and SKPs	➢ Pomote wound healing and functional tissue skin regeneration Chen et al. (2023a)
ADM	HaCaTs, Fibroblasts, HUVECs	➢ Promoted wound healing and re-epithelization, stimulated dermal ECM secretion and angiogenesis Jin et al. (2021)
HAMA	NHDFs, HFDPC	➢ Epidermal/papillary dermis and hair follicle structure improve cellular microenvironment and promote wound healing quality Kang and Kwon, (2022)
PEGDA	HaCaTs, Fibroblasts	➢ Promote the formation of epidermal layers with undulating microstructures and accelerate wound healing Shen et al. (2021)
Decellularized adipose tissue, HAMA	hADSCs	➢ Accelerate wound healing and improve healing quality by promoting angiogenesis Fu et al. (2023)
Nano-cellulose	Fibroblasts, HaCaTs	➢ Promote epidermis reconstruction and stratification; Reconstruction of skin with hair follicles and early reticular crest structure Li et al. (2023b)
Alginate	Fibroblasts, HaCaTs, HUVECs	➢ 3D skin model with layers of endothelial cell networks, dermal fibroblasts, and multilayered keratinocytes Barros and Kim, (2021)
Methacrylated silk fibroin	HaCaTs, Fibroblasts, HUVECs	➢ Mechanical stability and biocompatibility exceeding 4 weeks; enhance wound healing Choi et al. (2023)
Collagen, Tyrosinase	Melanocytes, HaCaTs, Fibroblasts	➢ Contribute to wound form epidermis and dermis Shi et al. (2018)
HA-NB	Fibroblasts, HUVECs	➢ Strong mechanical and bio-adhesive properties; promotes skin regeneration and efficient neovascularisation Zhou et al. (2020a)

Abbreviation: dHAM, decellularized human amniotic membrane; ADM, acellular dermal matrix; HAMA, hyaluronic acid methacryloyl; PEGDA, poly (ethylene glycol) diacrylate; HA-NB, butanamide linked hyaluronic acid.

Recent advances in 3D bioprinting technology have enabled the production of complex living 3D tissue analogues. GelMA is a promising candidate as a bioink for 3D printing of engineered skin tissues because of its excellent biocompatibility and tunable properties. Collagen and glycosaminoglycans are the two most abundant substances in the mammalian dermis (Papakonstantinou et al., 2012). To simulate the composition of collagen and glycosaminoglycan in natural skin, Kang et al. constructed tissue-engineered skin with hair follicle structures and papillary dermal layers using GelMA and hyaluronic acid methacryloyl (HAMA). GelMA and HAMA were used to recapitulate the composition of collagen and glycosaminoglycan in native skin, and hair follicle structures and papillary dermal layers were fabricated using 3D printing. Normal human dermal fibroblasts and hair follicle dermal papilla cell spheroids were embedded into GelMA/HAMA hydrogel scaffolds. HaCaTs were seeded on GelMA/HAMA hydrogel scaffolds. The printed hydrogel scaffolds exhibited appropriate degradation properties and a microporous structure similar to that of the native skin ECM. Furthermore, these GelMA/HAMA scaffolds showed the ability to induce hair growth and promote the spontaneous development of hair pores *in vitro* (Kang and Kwon, 2022). In another study, Chen et al. examined the effects of GelMA hydrogels combined with skin-derived precursors (SKPs) and epidermal stem cells (ESCs) on skin regeneration using an *in situ* robot-assisted bioprinting system. Their study demonstrated that GelMA hydrogels could not only serve as a scaffold for maintaining stem cell proliferation and the properties of SKPs but also showed promising potential in stem cell–based skin wound regeneration in mice. The regenerated skin was reported to contain epidermis, dermis, blood vessels, hair follicles, and sebaceous glands, similar to native skin (Chen et al., 2023a). Therefore, despite the availability of various implantable biomaterials and constructs for skin tissue engineering, the tunable mechanical properties and excellent cellular biocompatibility of GelMA hydrogels have significant implications in treating skin wound defects.

### 3.2 Role of GelMA hydrogels in wound dressings

Wound healing is a cascaded and highly complex process that primarily includes haemostasis, inflammation, proliferation, and remodelling. However, this biological process can be disrupted by bacterial infections, persistent inflammation, an insufficient blood supply, and excessive oxidative stress. These factors can delay wound healing, which may lead to non-healing chronic wounds. Therefore, dressings with anti-inflammatory, antibacterial, angiogenic, and antioxidant properties must be developed to promote wound healing. GelMA hydrogels loaded with various biological components and cytokines (such as nanoparticles, metal ions, and exosomes) are competitive candidates because of their excellent biological characteristics (Table 2).

**TABLE 2:** GelMA application in wound dressings.

Composite components	Dressing function	Property
AgBr@SiO_2_microspheres	Antibacterialdressings	➢ Enhanced mechanical properties, effective antimicrobial activity Li et al. (2022)
Electrospunnanofibers, dopamine	Joint wound dressings	➢ Excellent adhesive, breathable and stretchable capacities Liu et al. (2022)
C60@PDA	Antioxidative dressings	➢ Sustainable free radical scavenging ability, favorable cytocompatibility, and antibacterial ability Chen et al. (2023b)
Chitosan, glycerol, dopamine	Hemostatic sponges	➢ Good biocompatibility, tissue self-adhesion, antibacterial activity, and hemostatic ability Li et al. (2023c)
Pectin methacrylate	Hemostatic hydrogel	➢ Tunable rheology, highly porous structure, and controllable swelling and rapid crosslinking properties Wang et al. (2021)
Catechol	Hemostatic sealant	➢ Mussel-inspired hemostatic property and tissue adhesion Baghdasarian et al. (2022)
Acrylate, CuCl_2_	Diabetic wound dressings	➢ Efficient self-healing properties, antibacterial activity, and good adhesive properties Baghdasarian et al. (2022)
Desferrioxamine (DFO)	Diabetic wound dressings	➢ Controlled release of DFO and promote angiogenesis Chen et al. (2016)
VH298-EVs	Diabetic wound dressings	➢ Sustained release of VH-EVs enhances blood supply and angiogenesis Wang et al. (2022)
Dopamine, MSC-EVs	Diabetic wound dressings	➢ Rescue wound microenvironment homeostasis and accelerates wound closure Wang et al. (2023)
SFMA, MSN-RES, PDEVs	Diabetic wound dressings	➢ Sustained release of MSN-RES and PDEVs, regulate the inflammation and angiogenesis of diabetic wounds Zhu et al. (2022)
Mg^2+^, Zn^2+^	Skin wounds regeneration	➢ Sustained release Mg^2+^ and Zn^2,^ accelerate collagen deposition, angiogenesis and skin wound re-epithelialization Wang et al. (2022)
Hyaluronic acid-aldehyde, gentamicin sulfate, lysozyme	PH-responsive antibacterial dressings	➢ PH-responsive release drug with antibacterial and hemostatic effect Du et al. (2023)
Endothelin-1	Angiogenic dressing	➢ Accelerate angiogenesis and re-epithelialization Li et al. (2021)
HUVECs-EVs	Angiogenic dressing	➢ Sustained release of EVs, improve angiogenesis and wound healing Guo and Liu, (2022)
AFPBA, G-insulin	Microneedle dressing for diabetic wound healing	➢ Adequate mechanical properties, high biocompatibility, glucose-responsive insulin release Chen et al. (2022b)
PEGDA,HUVECs-derived exosomes, tazarotene	Microneedle dressing	➢ Promote cell migration, angiogenesis by slowly releasing exos and tazarotene in the deep layer of the skin Yuan et al. (2022)
PLGA,MSC	Microneedle dressing	➢ Effectively transport MSCs to target tissues and maintain high cell viability Lee et al. (2020)
Graphene oxide	Skin wound defects repair	➢ Accelerate vascularization of full-thickness skin defect Liang et al. (2022)
Silver, bFGF	Burn wounds dressings	➢ Sustained release of silver and bFGF, shorten the healing time of deep partial-thickness burn wounds Chen et al. (2022b)
nano silver	Skinwound defects repair	➢ Sustained release of nano silver, reducing wound exudation and promoting new tissue formation Jin et al. (2023)

Abbreviation: AgBr@SiO_2_, nanosized silver bromide-doped mesoporous silica; PDA, polydopamine; SFMA, silk fibroin glycidyl methacrylate; MSNs, mesoporous silica nanoparticles; PDEVs, platelet-derived extracellular vesicles; RES, resveratrol; AFPBA: glucose-responsive monomer 4-(2-acrylamidoethylcarbamoyl)-3-fluorophenylboronic acid; PLGA, poly(lactic-co-glycolic) acid.

#### 3.2.1 GelMA hydrogels promote wound angiogenesis

Wound healing requires blood to provide nutrients and oxygen for cell growth. The early recovery of the vascular network after injury is a key factor in preventing wound expansion and ulcer formation (Chen et al., 2016). Therefore, the development of angiogenic dressings is an important strategy for improving wound healing. Wound healing is a great challenge in diabetes due to poor angiogenesis and impaired cell function (Okonkwo and DiPietro, 2017; Aitcheson and Frentiu, 2021). GelMA hydrogels are ideal biomaterials for developing vascular networks because they provide a permissive environment for vascular morphogenesis. Chen et al. first demonstrated that the implantation of cell-laden GelMA hydrogels into immunodeficient mice resulted in the rapid formation of microvascular networks (Chen et al., 2012). Subsequently, several studies have shown that GelMA hydrogels can be used as excellent scaffold materials or drug-release carriers for the formation of microvascular networks. Li et al. prepared a multifunctional hydrogel dressing by encapsulating endothelin-1 (ET-1) in GelMA hydrogels for full-thickness wound healing. ET-1 is an endogenous vasoconstrictor that promotes angiogenesis in endothelial cells. GelMA hydrogels protected ET-1 from environmental damage and provided long-term promotion of the adhesion and proliferation of vascular endothelial cells via the sustained release of ET1. Animal experiments have shown that GelMA-ET-1 hydrogels significantly promoted the formation of new blood vessels and exhibited better wound healing on the 7th and 14th days (Li et al., 2021). Similarly, a desferrioxamine (DFO)-loaded GelMA hydrogel was developed for the rapid formation of vascular network structures in diabetic wounds. DFO could significantly accelerate the formation of new blood vessels by increasing the expression of HIF-1a and VEGF (Balanos et al., 2002; Li et al., 2014). In that study, DFO-GelMA hydrogels rapidly recruited angiogenesis-related cells and cytokines to the wound area and provided 3D microarchitectures for the formation of new blood vessels in the early stages of wound healing (Chen et al., 2016). GelMA hydrogels were also reported to be an ideal vehicle to preserve and deliver extracellular vesicles (EV) for *in vivo* vascularisation. In a previous study, epidermal stem cell–derived EVs loaded with VH298 were encapsulated in GelMA hydrogels to enhance the angiogenic ability of diabetic wounds. GelMA hydrogels were shown to be convenient and adaptable delivery carriers for the sustained release of VH298-EVs, effectively promoting wound healing by locally improving blood supply and angiogenesis by increasing the HIF-1a level (Wang et al., 2022). Similarly, EVs derived from human umbilical vein endothelial cells (HUVECs-EVs) were loaded onto 10% GelMA hydrogels to promote wound healing. GelMA-HUVECs-EV hydrogels could promote angiogenesis and skin regeneration via the sustained release of HUVECs-EVs during wound healing (Zhao et al., 2020).

#### 3.2.2 GelMA hydrogels inhibit bacterial growth and inflammation

Many adverse factors can interrupt the physiological healing processes. Among them, infection and inflammation are the most common, but moderate inflammation can promote wound healing by removing necrotic tissue and killing local bacteria (Almadani et al., 2021). Excessive inflammatory cell infiltration can hamper wound collagen deposition, angiogenesis, and granulation tissue formation (Huang et al., 2022). To improve the antibacterial function and mechanical properties of GelMA hydrogels, Li et al. prepared AgBr@SiO_2_/GelMA dressings by incorporating AgBr@SiO_2_ microspheres into GelMA solution and crosslinking it with UV light. The results indicated that AgBr@SiO_2_ microspheres not only improved the mechanical properties of GelMA hydrogels but also showed effective antibacterial activity against *Staphylococcus aureus* and *Escherichia coli* at a concentration of 1 mg mL^−1^. Treatment of full-thickness skin wounds in Sprague-Dawley rats with GelMA hydrogels containing 1 mg mL^−1^ AgBr@SiO_2_ significantly shortened the wound healing time and reduced the wound area (Li et al., 2022). Similarly, 3D bioprinting of GelMA hydrogels loaded with silver nanoparticles could improve full-thickness skin defect wound healing in rats by reducing wound exudation and promoting new tissue formation (Jin et al., 2023). Diabetic wounds are difficult to heal because of a wound microenvironment disorder caused by high glucose levels, which results in an extremely high risk of bacterial infection and a high state of oxidative stress. To promote the repair of diabetic wounds, Chen et al. prepared self-healing, adhesive, and antibacterial hydrogels using GelMA containing adenine acrylate (AA) and CuCl_2_. The coordination of hydrogen bonds and metal ligands provided by copper ions and carboxyl groups resulted in composite hydrogels exhibiting effective self-healing properties, significant fatigue resistance, and good adhesion properties. Among these, GelMA/AA/Cu1.0 hydrogels exhibited well-balanced biocompatibility and antibacterial properties and significantly promoted wound healing by inhibiting the expression of pro-inflammatory factors by releasing copper ions in full-thickness skin diabetic wounds (Chen et al., 2022a). Excessive ROS levels can also hinder the transition of the wound from the inflammatory to the proliferative stage, leading to a persistent inflammatory state. To improve wound healing, an ROS-scavenging hybrid hydrogel was designed by mixing a mussel-inspired fullerene nanocomposite (C60@PDA) dispersion into a GelMA solution with a concentration of 0.5 mg mL^−1^. The composite hydrogels exhibited sustainable free radical scavenging and antibacterial abilities *in vitro*. In a mouse full-thickness wound defect model, the composite hydrogels showed anti-inflammatory and anti-infection effects by downregulating the expression of IL-6 and TNF-α and upregulating the expression of TGF-β (Chen et al., 2023b). Furthermore, composite hydrogels composed of GelMA and silk fibroin glycidyl methacrylate were used to regulate the microenvironment of diabetic wounds via the sustained release of resveratrol (RES). In this study, RES was loaded into mesoporous silica nanoparticles (MSNs) and mixed into composite hydrogels as an anti-inflammatory and antioxidant agent. The wound model of diabetes mice showed that composite hydrogels could inhibit the expression of TNF-α and iNOS, promote the expression of anti-inflammatory factors TGF-β1 and Arg-1, and accelerate wound healing (Zhu et al., 2022).

#### 3.2.3 GelMA hydrogels facilitate wound haemostasis

Injectable hydrogels can be used as haemostatic adhesives in surgical wounds by fully filling and adhering to the wounds, especially for soft and brittle organs, where traditional surgical wound closure techniques are limited (Hickman et al., 2018; Wang and Yang, 2023). Injectable hydrogels exhibit a more effective haemostatic effect because of their injectability and porous structures. Additionally, gelatine sponges have been widely used as haemostatic materials because of their excellent blood absorption (Wang et al., 2020; Ebhodaghe, 2022). Therefore, as gelatine derivatives, photosensitive GelMA hydrogels have promising applications in wound haemostasis and closure. Wang et al. designed a new haemostatic hydrogel by combining pectin methacrylate (PECMA) and GelMA. The composite hydrogel was injectable and dual cross-linkable; it could be injected directly into the wound and rapidly cross-linked under the stimulation of calcium ions and UV. The highly porous network and dual cross-linkable properties of the PECMA/GelMA hydrogel allowed it to absorb blood rapidly and solidify rapidly. The PECMA/GelMA hydrogel synergised the haemostatic properties of calcium ions on PECMA, amine residues on GelMA, and highly porous networks to achieve rapid blood absorption and coagulation. The PECMA/GelMA hydrogels was shown to stop bleeding and reduce the coagulation time by 39% in a porcine skin bleeding mode (Wang et al., 2021). Hydrogel adhesion is crucial for controlling bleeding, particularly in wet environments. To improve the ability of hydrogels to adhere to wet tissue surfaces, Baghdasarian et al. synthesised a haemostatic double-crosslinked hydrogel, named gelatine methacryloyl-catechol (GelMAC) hydrogel, by covalently coupling gelatine with catechol motifs and methacrylate groups. The *in vitro* blood clotting assay showed that GelMAC significantly reduced the clotting time compared to the clinically used haemostat and Surgicel^®^. GelMAC exhibited good haemostatic properties and excellent tissue adhesion in a rat model (Baghdasarian et al., 2022). Recently, Li et al. developed a composite sponge using dopamine-modified GelMA, quaternised chitosan (QCS), and glycerol (Gly). They found that modifying GelMA with dopamine enhanced the self-adhesion of the composite sponges. Animal experiments have shown that both the haemostasis time and blood loss in the GelMA-DA/QCS/Gly sponge group were lower than those in the commercial gelatine haemostatic sponge and the haemostatic sponge CS (Li et al., 2023c). These results demonstrate the broad application prospects of GelMA-based biomaterials as haemostatic wound dressings in clinical surgery and emergency treatment.

### 3.3 GelMA hydrogel microneedles patch for wound healing

Microneedles are one of the transdermal drug delivery techniques that usually release loaded drugs directly into the deep layer of the skin through tiny holes formed by puncture (Donnelly et al., 2010). Hydrogel microneedles have gradually become popular because of their convenient administration and few side effects (Waghule et al., 2019). GelMA microneedles exhibit high potency for subcutaneous micro-invasive transdermal targeted drug delivery because of their adjustable mechanical properties and swelling capacity (Zhou et al., 2020b; Fonseca et al., 2020). Subcutaneous insulin injection is a typical clinical solution for diabetes. To avoid adverse reactions caused by the low controllability of subcutaneous insulin injections, a glucose-responsive insulin-releasing hydrogel microneedle dressing was fabricated. The microneedle dressing was composed of GelMA, glucose-responsive monomer 4-(2-acrylamidoethylcarbamoyl)-3-fluorophenylboronic acid, and gluconic insulin. The hydrogel microneedles showed sufficient mechanical properties, excellent biocompatibility, and glucose-responsive insulin release behaviour and were shown to be effective in diabetic wound management (Guo and Liu, 2022). The microneedle technology allows drugs to be controlled and released deep into the skin. To improve the drug delivery efficiency, a GelMA-based microneedle patch loaded with HUVECs exosomes and tazarotene was designed to accelerate diabetic wound repair (Yuan et al., 2022). These microneedle patches showed good performance in maintaining the biological activity of exosomes and drugs *in vitro*, achieving controlled and transdermal release in a mouse model of diabetes. The controlled release of drugs and HUVEC exosomes deep into diabetic wounds promoted cell proliferation, migration, and angiogenesis. Lee et al. constructed a detachable hybrid microneedle system for mesenchymal stem cell (MSC) delivery (Lee et al., 2020). The microneedle system was composed of polylactic acid hydroxyacetic acid as the outer shell and a GelMA-MSC mixture as the inner shell. After 24 h of microneedle preparation, cell viability remained above 90%, and mice treated with this microneedle system showed good wound recovery.

## 4 Challenges and future perspective

In conclusion, GelMA hydrogels have been widely used in many applications, ranging from wound dressings to 3D printing skin tissue engineering. They provide an ideal multistratified anisotropic scaffold for the growth of various cells such as fibroblasts, endothelial cells, and keratinocytes. They can also be used to prepare personalised multifunctional dressings by combining small molecules, metal nanoparticles, and cellular EVs via physical binding or chemical reactions. Therefore, the application prospects of GelMA hydrogels for wound healing are significant. However, the biosafety of GelMA-based hydrogels remains a major obstacle to their clinical applications. The release of unreacted methacryloyl monomers after photo-crosslinking is a potential risk. Furthermore, photo-crosslinking under UV radiation may damage cellular DNA (Matsumura and Ananthaswamy, 2004). Additionally, thorough and objective investigations on the cytotoxicity or biological safety of photoinitiators, nanocomposites, or metal ions incorporated into GelMA hydrogels are essential for future studies (Sakr et al., 2022; Ghazali et al., 2023). Therefore, the development of a milder and more efficient GelMA hydrogel crosslinking process is necessary for clinical application. Moreover, the properties and performance of GelMA hydrogels can be influenced by many factors, such as the degree of substitution of methacryloyl groups, amount of photoinitiator used, and photo-crosslinking conditions (Yue et al., 2015; Rajabi et al., 2021). The lack of a unified and precise preparation standard or process for most studies limits the reproducibility of GelMA preparations for biomedical applications and leads to inconsistent results. For example, in three different studies on the effects of GelMA hydrogels on wound angiogenesis, the concentrations of GelMA hydrogels used were 5%, 10%, and 15% (Chen et al., 2016; Li et al., 2021; Guo and Liu, 2022). Therefore, more detailed research is necessary to validate GelMA hydrogels for clinical applications.
